# Misrepresentation of Evidence Favoring Early Normalizing Surgery for Atypical Sex Anatomies

**DOI:** 10.1007/s10508-015-0529-x

**Published:** 2015-03-26

**Authors:** Arlene B. Baratz, Ellen K. Feder

**Affiliations:** 1 Androgen Insensitivity Syndrome—Disorders of Sex Development Support Group, P.O. Box 2148, Duncan, OK 73534 USA; 2 Department of Philosophy and Religion, American University, 4400 Massachusetts Ave. NW, Washington, DC 20016 USA

Questions concerning the timing of normalizing surgery in cases of atypical genitalia have been contentious ones, with pediatric specialists (and often parents) preferring early surgery, and critics arguing for deferral of decisions. Early surgery is intended to secure children the benefits of “normal appearance” and has been assumed important for a child’s psychosocial development. There is only anecdotal evidence on the question of the timing of surgery and this evidence has been employed both to support and to challenge the assumption of the benefits of early surgery.

In the absence of good evidence for the benefits of early surgery or of the harm of deferring surgery, Heino Meyer-Bahlburg, a proponent of the current standard of care, claims that evidence of opinions gathered from affected individuals themselves bolsters physicians’ long-standing preference for early surgery. In a chapter from the newly published edited volume, *Genetic Steroid Disorders*, Meyer-Bahlburg (2014) writes that:

Given the many examples of unsatisfactory outcomes, some DSD activists and ethicists have demanded a delay of any genital surgery until the age of consent (Greenberg, 2006). It is important, therefore, to note that in the three studies that have included survey questions on this matter, the majority of women with CAH have clearly favored genital surgery at an early age ([citing] Nordenskjöld et al., 2008; Wisniewski, Migeon, Malouf, & Gearhart, 2004; Minto, Liao, Woodhouse, Ransley, & Creighton, 2003), as have patients with other DSD syndromes ([citing] Meyer-Bahlburg et al., 2004). One study analyzing in detail for age effects found no significant correlations between age at surgery and ratings of genital cosmesis or function, except that the women reported higher satisfaction with clitoral function the earlier their genital surgery had been performed ([citing] Johannsen et al., 2010). (p. 295)

We are accustomed to taking academic researchers at their word, but Meyer-Bahlburg’s claims here require closer examination.

First, regarding the claim that “the majority of women…have clearly favored genital surgery at an earlier age,” it may be worth noting that none of the women to whom the question was posed in the studies cited appear to have been informed that (1) not having surgery at all might be an option; (2) they might have had lower rates of reoperation for stenosis if surgery were performed later, or (3) that significant technical improvements that were expected to improve outcomes had occurred in the 13 or 14 years between when they underwent early childhood surgery and when it might have been deferred until after puberty. The significance of the responses to the questions asked, in other words, should be assessed with respect also to relevant questions that were not asked as well as to potential lack of information provided to those questioned.

The claim that recent studies have demonstrated that “the majority of women with CAH have clearly favored genital surgery at an early age” is one that should be taken seriously. However, a closer look at each study suggests that Meyer-Bahlburg’s characterizations of these studies are misleading.

The first source Meyer-Bahlburg cites, “Psychosexual outcome in women affected by congenital adrenal hyperplasia due to 21-hydroxylase deficiency” (Wisniewski et al., 2004), involved contacting 41 women with CAH from the same clinic (Johns Hopkins) to participate in a survey on optimal timing for feminizing genitoplasty. Of those 41, 30 % did not respond. Of respondents who did answer, 40 % chose infancy or toddler age, but 30 % gave answers that divided among elementary school age, adolescence, and adulthood.

The second study, “Type of mutation and surgical procedure affect long-term quality of life for women with congenital adrenal hyperplasia” (Nordenskjöld et al., 2008), draws on a sample of 62 Swedish women with CAH. Only 29 responded to a question on timing of surgery. Of the 29 who responded, 20 preferred “early surgery,” while 9 “advocated surgery at puberty when proper information can be given.” Again, a significant proportion—one-half—did not answer this question, and one-third of those who did respond specifically preferred deferral until a developmental stage in which they would be able to understand relevant information. The women in the study noted that information they were given focused on “lifelong medication,” and they would have benefitted from information about gynecological, surgical, and psychosexual issues. Had they had the sort of information reported by these researchers regarding outcomes of surgery in the subject group—including “scarred and irregular tissues [in the clitoral region and labia minora], in addition to the non-anatomical size and location of clitoris”—one wonders if responses to the question concerning timing of surgery may have reflected a preference for deferral.

And finally, the third study, “The effect of clitoral surgery on sexual outcome in individuals who have intersex conditions with ambiguous genitalia: A cross-sectional study” (Minto et al., 2003), provides no data about women with CAH favoring earlier surgery. Questions addressing preference of adults for early surgery were not included in the study. In addition to the absence of evidence of a preference for early surgery by participants, this study challenges the presumed benefits of genitoplasty and substantiates the common sense understanding that genital surgery constitutes a harm. Minto et al. decry the absence of evidence supporting the claim that “feminizing genital surgery leads to better psychosocial outcomes than leaving the genitalia unaltered.” While normalizing interventions may seem “a logical and obvious course of action” to prevent (projected psychological) harm to children with atypical sex anatomies, Minto et al. reported that their findings provided evidence of significant harm to sexual function, and emphasized that decisions about such procedures are not simply “medical” issues, but “moral” issues.

Finally, Meyer-Bahlburg (2014) cited an earlier, fourth study he co-authored, from Johns Hopkins Hospital (Meyer-Bahlburg et al., 2004), that he represents as confirming the claim of the previous three. Of the 40 patients with XY chromosomes assigned female, 18 had “ambiguous genitalia,” 5 had “micropenis,” and the others had typical-appearing genitalia. Here is the question, regarding timing of surgery, posed to the 18 women with “ambiguous genitalia” (14 of whom—78 %—responded to the question):

“Some people argue that children born with unfinished sex organs (ambiguous genitalia) should not be surgically corrected before they are adult and can fully understand and consent to the procedures. Do you agree?: 1 Agree 13 Do not agree.” (Meyer-Bahlburg et al., 2004)

Characterizing genital ambiguity as a problem of “unfinished sex organs” would appear to be directive, promoting a bias in responses.

In the same study (Meyer-Bahlburg et al., 2004), posing a different question, gave what may be a more nuanced picture of respondents’ beliefs about timing of surgery. Of 15 women (83 %) who answered: “If you have had any operations on your sex organs: by hindsight, do you feel these operations should have occurred”: 1 answered “not before adulthood,” 6 answered “not before adolescence,” 1 answered “not before elementary school age,” 1 answered “not before toddler age,” and 6 answered “during infancy.” We should ask: Does 7 of 15 choosing adolescence and adulthood over childhood constitute the ringing endorsement of early surgery Meyer-Bahlburg’s citation supposedly indicates?

Incidentally, in this study (Meyer-Bahlburg et al., 2004), those assigned male were more evenly split on the first question: “Some people argue that children born with unfinished sex organs (ambiguous genitalia) should not be surgically corrected before they are adults and can fully understand and consent to the procedures. Do you agree?” Among those assigned male, 10 agreed that surgery should be postponed and 14 disagreed.

Regarding the claim that “women reported higher satisfaction with clitoral function the earlier their genital surgery had been performed,” Meyer-Bahlburg (2014) cited a more recent Danish study, “Long-term gynecological outcomes in women with congenital adrenal hyperplasia due to 21-hydroxylase deficiency” (Johannsen et al., 2010). In this study of 27 women with CAH who had had clitorectomies or clitoroplasty, they reported only that, “No significant correlations were found between age at surgery and rating of genitalia (data not shown), except a higher satisfaction with clitoral function the younger the age at surgery (*rs* = −.45, *p* = .03).”

Johannsen et al. (2010) did not present information about the response rate or how responses varied with age at surgery. It is possible, perhaps likely, that those operated on later were less satisfied as adults because they had, literally, a sense of their clitoral function preceding surgery. Johannsen et al.’s identification of satisfaction with early surgery as opposed to later surgery could appear to confirm the inverse conclusion, namely, the one that accords with the common-sense correlation of decreased erotic sensation with dissatisfaction with clitoral function.

It may not be surprising that research, and perhaps especially research into areas that medicalize what might be regarded as social problems, is vulnerable to bias in a variety of ways. As discussed here, closer evaluation of actual research methods and data can expose flawed and misleading conclusions that require more extensive interpretation and critical debate. Especially when an author is esteemed in a field, claims about a controversial issue that are presented as well-grounded are often reproduced repeatedly and unquestioningly. When such claims support the genuine hopes of physicians and parents for a “fix” despite existing evidence of the considerable risks of harm, a more critical review of existing research is in order. Avoiding these pitfalls requires inclusion of patient advocates from the very earliest stages in designing the goals and methods of future research.
